# Evaluation of *Pontederia crassipes* as bioindicator of heavy metals in Lake Manzala, Egypt

**DOI:** 10.1038/s41598-026-49783-7

**Published:** 2026-05-07

**Authors:** Samah Ramadan, Maha M. Elshamy, Elsayed M. Nafea

**Affiliations:** 1 Botany Department, Faculty of Science, Mansoura University, Mansoura, 35516 Egypt; 2 Aquatic Environment Department, Faculty of Fish Resources, Suez University, Suez, Egypt

**Keywords:** *Pontederia crassipes*, Water pollution, Heavy metals, Bioaccumulation, Translocation factor, Ecology, Ecology, Environmental sciences

## Abstract

Heavy metals are among the most critical pollutants affecting aquatic ecosystems due to their persistence, toxicity, and bioaccumulation potential. Lake Manzala, the largest coastal lake in Egypt, is increasingly exposed to contamination from agricultural, industrial, and domestic discharges. This study evaluated the potential of *Pontederia crassipes* (water hyacinth) as a bioindicator and phytoremediator of heavy metals in three locations of Lake Manzala. Concentrations of iron (Fe), zinc (Zn), copper (Cu), lead (Pb), nickel (Ni), and cadmium (Cd) were determined in water and plant tissues using inductively coupled plasma–atomic emission spectrometry (ICP-AES). Bioaccumulation was assessed using the Biological Accumulation Coefficient (BAC), Bioconcentration Factor (BF), and Translocation Factor (TF). The results showed that metal concentrations in water followed the order: Fe > Zn > Cu > Pb > Ni > Cd, with significantly higher levels (p < 0.05) recorded at the northeastern site. *P. crassipes* accumulated all metals predominantly in roots, with significantly higher concentrations than in leaves (p < 0.05). BF values exceeded 1 for all metals, indicating strong accumulation capacity, while TF values remained below 1, suggesting limited translocation to aerial parts. Significant positive correlations (p < 0.05) were observed between metal concentrations in water and plant tissues. These findings demonstrate that *P. crassipes* is an effective bioindicator and phytostabilizer of heavy metals in freshwater ecosystems. The study highlights its potential application in environmental monitoring and sustainable phytoremediation strategies for polluted aquatic ecosystems such as Lake Manzala.

## Introduction

The pollution of aquatic ecosystems by heavy metals has become a major global environmental concern due to their persistence, non-biodegradability, and potential for bioaccumulation in food chains^1,2^. Anthropogenic activities such as industrial discharge, agricultural runoff, and domestic wastewater release substantial quantities of toxic metals into freshwater systems, posing serious risks to ecological integrity and human health^3–5^. Even at low concentrations, heavy metals can exert toxic, mutagenic, and carcinogenic effects on aquatic organisms and humans, affecting vital organs and disrupting biological processes.

Coastal lakes in Egypt, particularly those located along the Mediterranean coast, are highly vulnerable to pollution due to increasing urbanization and agricultural expansion. Among these, Lake Manzala is crucial for Egypt’s fishing industry, accounting for 36–50% of the nation’s yearly fish production due to its large fishing community of 15,975 licensed fishermen and 2,785 fishing boats^6,7^. However, the lake has undergone severe environmental degradation in recent decades, primarily due to the continuous discharge of untreated agricultural, industrial, and domestic wastewater. These inputs have altered water quality, reduced biodiversity, and led to the accumulation of contaminants, including heavy metals, in the aquatic environment^8–10^.

Phytoremediation has emerged as an environmentally friendly and cost-effective approach for mitigating heavy metal pollution in aquatic environments. This technique utilizes plants to remove, stabilize, or detoxify contaminants through mechanisms such as phytoextraction, phytostabilization, and rhizofiltration^11,12^. Several aquatic macrophytes, including *Pistia stratiotes*, *Lemna minor*, and *Typha* spp., have been widely investigated for their capacity to accumulate heavy metals and improve water quality^13^^,^^14^.

Among these species, *Pontederia crassipes* (water hyacinth) has attracted considerable attention due to its rapid growth rate, high biomass production, extensive fibrous root system, and remarkable capacity to accumulate heavy metals^15–17^. Its roots provide a large surface area for adsorption and absorption of metal ions, making it particularly effective in contaminated aquatic environments. Additionally, its widespread distribution and abundance in Egyptian freshwater systems make it a practical candidate for large-scale phytoremediation applications^18–22^. These characteristics also support its use as a bioindicator, as its tissue metal concentrations often reflect ambient environmental conditions^23–26^.

In lakes, canals, and drains, the species *Pontederia crassipes* is represented and has grown to become one of the dominant free-floating hydrophytes^27–30^. *Pontederia crassipes* (previously *Eichhornia crassipes*), belonging to the family Pontederiaceae, is usually referred to as common water hyacinth. It is indigenous to South America, has naturalized globally, and frequently exhibits invasive characteristics beyond its original habitat^31^. *P*. *crassipes* can be utilized to bioremediate and purify water, improving the quality of drinking water by removing various pollutants. Its roots and leaves can also be utilized as bio filters for heavy metals^32,33^. Hydrophytes greatly favor the improvement of water quality in contaminated areas^34^. Due to its environmental plasticity, the plant was found in a variety of territories in Egypt, including freshwater, brackish, salt-influenced wetlands, and even coastal drain habitats^18^.

Bioaccumulation factor (BF) is a calculated value that indicates the ability of plants to remove metals from the soil/water. On the other hand, translocation factor (TF) refers to the ability of a metal to move from plant roots to other organs^4,21^. Plants with BF greater than 1 can be used as bioaccumulators^35^. A BF greater than 2 is considered a high value^35^. Plants can be used as phytostabilizers if the BF is greater than 1 and the TF is less than 1, and as phytoextractors if the BF is less than 1 and the TF is greater than 1^36^.

Despite the extensive body of research on phytoremediation using *P. crassipes*, several knowledge gaps remain. Most previous studies have focused either on controlled experimental conditions or on single-site assessments, with limited emphasis on spatial variation within large, heterogeneous aquatic systems. Furthermore, few studies have simultaneously evaluated the relationships between water metal concentrations and plant accumulation using integrated indices such as Biological Accumulation Coefficient (BAC), Bioconcentration Factor (BF), and Translocation Factor (TF). In the context of Lake Manzala, comprehensive studies that combine spatial assessment of heavy metal distribution with plant-based bioindication and phytoremediation potential are still limited. Therefore, the novelty of the present study lies in (i) the simultaneous assessment of heavy metal distribution in water and different organs of *P. crassipes* across multiple sites with varying pollution levels within Lake Manzala, (ii) the integrated evaluation of BAC, BF, and TF to characterise accumulation and translocation behaviour, and (iii) the dual application of *P. crassipes *as both a bioindicator and a phytoremediator under natural field conditions in a highly impacted deltaic lake. Accordingly, the objectives of this study were to (i) investigate the spatial distribution of heavy metals in water and plant organs of *P. crassipes*; (ii) evaluate the accumulation capacity of different plant organs using BAC and BF; and (iii) assess the suitability of *P. crassipes* as a bioindicator and phytoremediation agent in Lake Manzala.

## Materials and methods

### Study species

*Pontederia crassipes* (water hyacinth) is a free-floating, perennial aquatic plant characterized by large, thick, shiny, ovate leaves and can grow up to 1 meter above the water’s surface. Its leaves are 10–20 cm in diameter on a stem that floats due to buoyant, bulbous nodules at its base, positioned above the water’s surface. The pendulous, fibrous roots are a shade of purple black. A vertical stem sustains a solitary spike with 8–15 visually striking blooms, predominantly lavender to pink, including six petals^37^. Water hyacinth, recognized as one of the most rapidly proliferating plants, typically reproduces by runners that ultimately generate daughter plants. Moreover, each plant can produce thousands of seeds each year, which may stay viable for over 28 years. *P*. *crassipes* exhibit rapid growth, with mats capable of doubling in bulk within one to two weeks. Regarding plant quantity instead of dimensions, it is reported that they can increase by almost a hundredfold within 23 days^38^.

### Study site

**Fig. 1:**
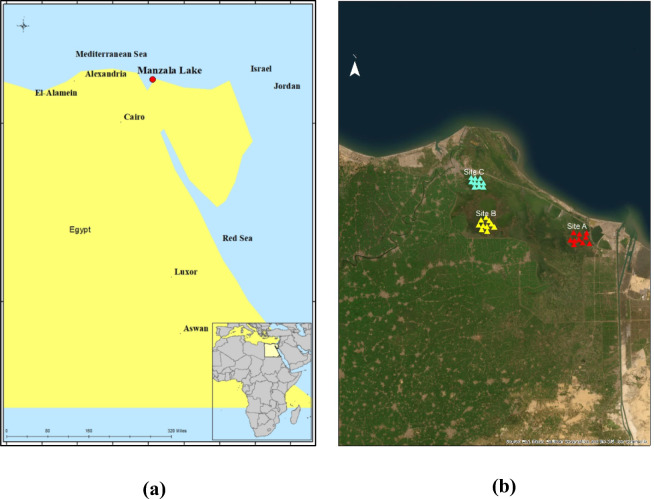
**(a)** Geographic location of Lake Manzala in Egypt, with an inset map showing the position of Egypt within Africa. **(b)** Study area map of Lake Manzala showing the sampling sites (Sites A, B, and C). The figure was prepared by the authors using ArcMap 10.7 (Esri, Redlands, CA, USA), available at https://www.esri.com/en-us/arcgis/products/arcgis-desktop/overview

Lake Manzala lies on the northeastern boundary of the Nile Delta. It is located between latitudes 31°10′ and 31°40′N and longitudes 31°50′ and 32° 25′E^39^. It adjoins the Mediterranean Sea to the north. The governorates of Ismailia and Port Said, which border the lake on its eastern side, border the Suez Canal. Al-Sharqiya governorate borders the lake from the north, while the southwest surrounds Dakahlia governorate. The lake borders the western portion of the Damietta Governorate. Egypt boasts a semi-arid climate, marked by hot and humid summers, moderate winters, and minimal rainfall. The climatic conditions are diverse in the lake study region, with an average air temperature ranging from 16 degrees Celsius in January to 29 degrees Celsius in August. There is a minimal amount of rainfall, typically less than fifty millimeters, from December to February. There is a range of wind speeds from 10 to 40 kilometers per hour (http://meteo.infospace.ru/climate/html/, https://www.wunderground.com/ ).We conducted the current study at the northeastern (site A), middle (site B), and northwestern parts of Lake Manzala (site C) (Fig. 1).

### Plant and water sampling

Leaf and root samples of *Pontederia crassipes* were collected from the studied sites in Lake Manzala during spring 2022, with ten plant samples taken from each site. Whole plants were gathered randomly from the littoral zone using clean gloves and stainless-steel scissors to ensure both leaves and roots were included. The collected specimens (Authentication number: Pcr3672) were washed thoroughly with distilled water, air-dried, and placed in the Herbarium of the Faculty of Science at Mansoura University. The species was identified according to Loutfy Boulos^28^ based on its morphological characteristics, with assistance from Maha M. Elshamy, a Professor of Plant Taxonomy at the Faculty of Science, Mansoura University.

Plant samples were collected from their natural habitats using methods that respected the environment and protected the plant populations. Fieldwork and specimen collection followed institutional, national, and international guidelines, including those from the International Union for Conservation of Nature on research involving threatened species and the Convention on International Trade in Endangered Species of Wild Fauna and Flora regulations. Ten plant samples were collected from each site, resulting in a total of 30 plant samples across the three study sites. At the same time, water samples were taken from a depth of 0.5 to 1 meter using a pre-cleaned water sampler and placed into acid-washed polyethylene bottles. Ten water samples were collected per site, yielding a total of 30 water samples. Sampling locations within Lake Manzala were selected based on the presence of *P. crassipes* and differing pollution levels, determined by their proximity to drainage inputs and established pollution gradients.

### Physicochemical water analysis

The pH and temperature (°C) were measured using a Misuraline ML 1010 meter. Water depth (m) and transparency were determined using a Secchi disc. The electrical conductivity (EC) (mmhoS/cm) and total dissolved salts (TDS) (mg/L) were determined using an electronic meter (HANNA, model HI 99300). With the aid of HANNA’s model HI 9146, dissolved oxygen (DO) (mg/L) was determined^40^, biological oxygen demand (BOD) was determined following APHA^41^. The molybdate blue technique was used to analyze the soluble reactive phosphate (PO_4_^2-^)^42^. The chromic acid method was utilized to determine the nitrate levels (NO_3_^-^). Filtered water samples are placed in an aliquot and treated with sodium salicylate. The Griess-Ilosvary method was modified to determine nitrite (NO_2_^-^). Nessler’s method, that uses alkaline mercury chloride solution as a reagent for the colorimetric investigation, was used to determine ammonia levels. Reactive silica (SiO_3_^2-^) was analyzed according to molybdenum silicate method described in APHA^43^. Acid molybdate reagent (3 ml) was added to 50 ml of the sample, then after 10 min 5 ml of (50%) sulfuric acid was mixed with them. oxalic acid (2 ml, 10%) was added within 5 min stannous chloride reagent (1ml) was added. Finally, the absorbance of the mixture was measured, within 20-40 min, (using spectrophotometer, model 21D) at 690 nm using distilled water as a blank. The concentration of SiO_3_^2-^ of water samples was calculated from the calibration curve.

### Heavy metals in water and plant parts

Water samples were acidified instantly with nitric acid in the field for heavy metals determination. The plant samples were subjected to a 48-hour drying process at 72° C, after which the leaves and roots were extracted and subsequently pulverized into a fine powder. Subsequently, samples were subjected to digestion in accordance with the methodology outlined by Temmingho and Houba^44^. ICP-AES (inductively coupled plasma-atomic emission spectrometry,Ultima2 JY, Horiba Company, Edison, NJ, USA) was employed to assess metal concentrations.

### Biological accumulation coefficient (BAC), bioconcentration factor (BF), translocation factor (TF)

The Biological Accumulation Coefficient (BAC) denotes the proportion of an element’s concentration in the biological system to element overall concentration in the water^45^ and is determined using this equation: BAC Cplant leaf/Cwater,Cplant leaf and Cwater denote heavy metals’ concentrations in the plant leaves and water, correspondingly.

The bioaccumulation factor (BF) is a metric that measures a plant’s ability to accumulate a specific metal in relation to its concentration in water^45^, was computed as: BF = Cplant root/Cwater,Cplant root and Cwater denote the concentrations of heavy metals in the plant root and water.

We evaluated the translocation factor (TF) to determine the relative translocation of metals from the subterranean root to the aerial parts of the plant species^46^. It was computed as TF = Cleaf/Croot,Cleaf is heavy metal concentration in the plant leaves and Croot denotes heavy metal concentration in the plant root.

### Data analysis

One-way analysis of variance (ANOVA), followed by Tukey’s Honestly Significant Difference (HSD) post hoc test, was performed to assess significant differences in water physico-chemical parameters and heavy metal concentrations (p < 0.05) across the three sampling sites. The statistical software used for this analysis was SPSS version 26. The GraphPad Prism 9.0.2 software (GraphPad Software, Inc., La Jolla, California, United States) was used to do all the figures, including Pearson correlations and regression analysis.

## Results

### Physico-chemical parameters of water

The physicochemical properties of Lake Manzala during Spring 2022 are summarized in Table 1, with values expressed as mean ± SE. Significant differences among sites were observed for most parameters (p < 0.05), except for temperature, pH, and SiO₃^2^⁻, which did not differ significantly among sites (Table 1). All investigated physico-chemical parameters, except temperature, pH and SiO_3_^2-^ were differed significantly (Table 1). The temperature reached between 30.80**±**0.74 and 31.60**±**0.51. Water pH was found to be a little basic, ranging from 7.84**±**0.18 to 8.76**±**0.26. Site A had the maxium temperature (31.60**±**0.60 °C) and transparency (80.60**±**7.06 cm), while site B had the lowest depth, temperature and transparency (127.00**±**20.83cm, 30.80**±**0.74 ^°^C and 52.00**±**8.46 cm). Meanwhile, site C recorded the highest depth (217.00**±**7.68 cm).EC greatly ranged between the values 1.48**±**0.29 and 19.40**±**2.38 mmhoS/cm). Total Dissolved Solids (TDS) also varied widely along the lake, reached maximum value (8.86**±**2.53 mg/L) at site A and minumum value (0.90**±**0.08 mg/L) at site C (Table 1).

**Table 1:** Physicochemical properties of water in Lake Manzala at the study sites (A, B, C).

**Parameter**	**Site A**	**Site B**	**Site C**	**F- value**
Depth (cm)	193.00±11.79^b^	127.00±20.83^a^	217.00±7.68^b^	10.31*
Temp. (^°^C)	31.60±0.51^a^	30.80±0.74^a^	31.00±0.55^a^	0.47^ns^
Transparency (cm)	80.60±7.06^b^	52.00±8.46^a^	57.40±4.93^a^	4.76*
pH	7.84±0.18^a^	7.94±0.47^a^	8.76±0.26^a^	2.38^ns^
EC (mmhoS /cm)	19.40±2.38^b^	3.20±0.58^a^	1.48±0.29^a^	48.24*
TDS (mg/L)	8.86±2.53^b^	1.64±0.14^a^	0.90±0.08^a^	9.06*
DO (mg/ L)	3.49±0.68^b^	7.36±0.50^a^	8.14±0.49^b^	15.95*
BOD (mg/ L)	6.02±0.16^a^	2.86±0.76^a^	2.46±0.37^b^	15.49*
PO_4_^2-^ (mg/ L)	15.50±0.19^a^	2.78±1.58^b^	1.29±0.47^a^	62.88*
NH_4_^+^ (mg/ L)	14.50±0.21^a^	1.80±1.79^b^	1.58±0.17^a^	50.16*
NO_3_^-^ (mg/ L)	9.30±0.18^a^	1.94±0.17^a^	1.42±0.52^b^	178.73*
NO_2_^-^ (mg/ L)	9.92±0.07^a^	1.48±0.29^a^	1.14±0.91^b^	80.55*
SiO_2_^3-^ (mg/ L)	28.20±4.14^a^	26.08±2.99^a^	24.00±1.92^a^	0.44^ns^

Values represent mean ± SE (n = 10). Different superscript letters within the same row indicate significant differences among sites according to Tukey’s test (P < 0.05). ns = not significant; *P < 0.05.

The high concentrations of DO (8.14**±**0.49, 7.36**±**0.50 mg/L) were at site C and site B, respectively. Conversely, site A exhibited the most BOD concentrations (6.02**±**0.16 mg/L). Furthermore, the highest concentrations of PO_4_^3-^, NH_4_^+^, NO_3_^-^, and NO_2_^-^ (15.50**±**0.19, 14.50**±**0.21, 9.30**±**0.18, and 9.92**±**0.07 mg/L) were recorded at site A. Silicate ions (SiO_2_^3-^) reached maximum values (28.20**±**4.14 mg/L) at sit A, while minimum values (24.00**±**1.92 mg/L) at site C (Table 1).

**Fig. 2:**
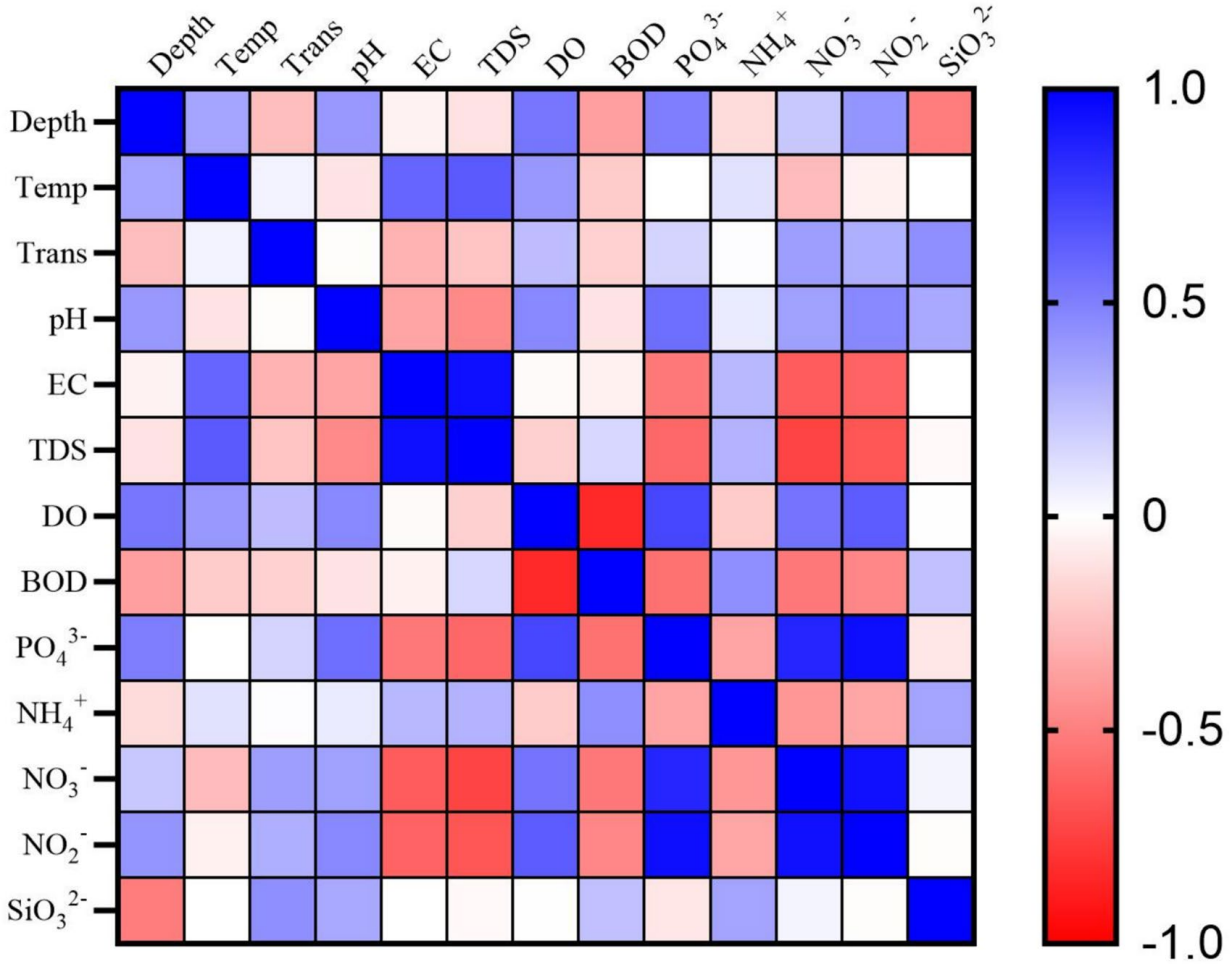
Heatmap of Pearson correlation coefficients among selected physicochemical parameters in Lake Manzala. Color gradients represent the strength and direction of correlations, with blue indicating positive correlations and red indicating negative correlations. Temp: Temperature; Trans: Transparency; EC: Electrical Conductivity; TDS: Total Dissolved Salts; DO: Dissolved Oxygen; BOD: Biological Oxygen Demand; PO_4_^2-^ : Phosphate; NH_4_^+^: Ammonium; NO_2_^-^ : Nitrite, NO_3_^-^: Nitrate; SiO_3_^-^: Silicate.

The correlation matrix (Fig. 2) indicated that pH has a strong positive association with PO_4_^3-^, DO and NO_2_^-^, while demonstrating a negative correlation with TDS and temperature. EC had a positive association with TDS and temperature, while demonstrating a negative correlation with NO_3_^-^, NO_2_^-^ and PO_4_^3-^. A notable positive association was identified among DO, PO_4_^3-^, NO_2_^-^ and NO_3_^-^. A significant negative connection (P < 0.05) was identified between BOD and DO.

### Heavy metals in water

**Fig. 3:**
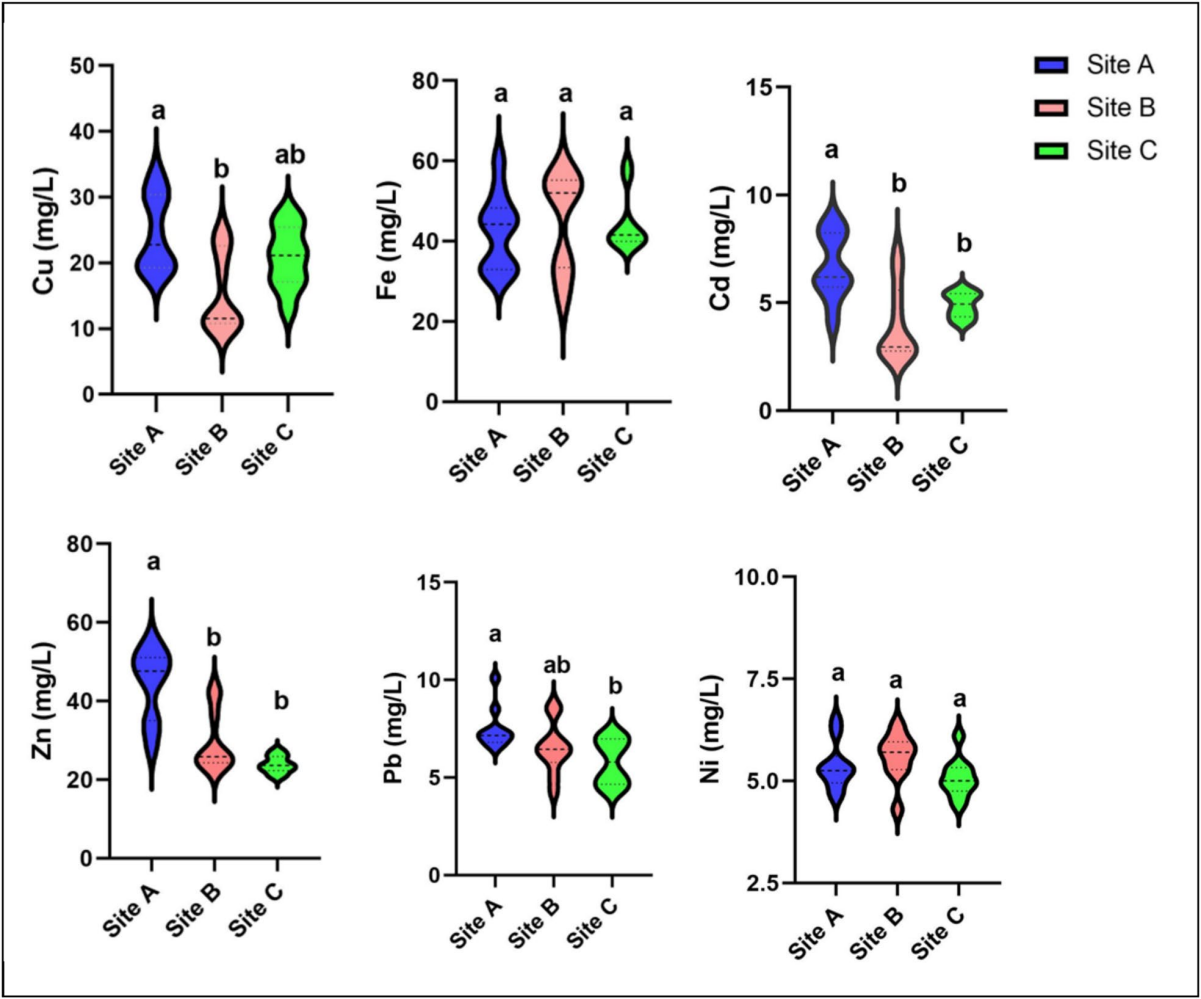
Violin plots show heavy metals concentrations in water at different sites along Lake Manzala (in milligrams per liter). Different superscript letters indicate significant differences among sites according to one-way ANOVA followed by Tukey’s post hoc test (P < 0.05).

Cu, Fe, Cd, Zn, Pb, and Ni were chosen for analysis because of their environmental significance, toxicity, and frequent occurrence in aquatic ecosystems impacted by human activity. Cu, Fe, Cd, Zn, Pb, and Ni concentrations in water samples varied from 10.4–33.6, 25.3–60.1, 2.6–8.8, 20.6–55.3, 4.3–10.1, and 4.3–6.5 mg/L, respectively (Fig. 3). Site A had the highest concentrations of all the metals that were examined, while site C had the lowest values.

Cadmium (Cd) had the lowest mean concentration (5.2 mg/L) among the metals under investigation, while iron (Fe) had the highest mean concentration (43.9 mg/L). The order of the mean metal concentrations in water samples was Fe > Zn > Cu > Pb > Ni > Cd. Anthropogenic inputs, such as agricultural drainage, household wastewater discharge, and industrial effluents entering Lake Manzala through multiple drainage canals, may be responsible for the elevated concentrations of these metals. Heavy metal buildup in the lake’s water column is known to be greatly influenced by such inputs.

### Plant heavy metals

The data presented in Table 2 show changes in heavy metal levels between the roots and leaves of *P. crassipes*. The results indicate that the concentrations of Cu, Fe, Cd, Zn, Pb, and Ni in the roots varied significantly (p < 0.05) across different tissue locations. In the leaves, all analyzed metals except Fe also exhibited significant variation among the studied sites. Overall, *P. crassipes* accumulated higher concentrations of all heavy metals in its roots than in its aerial tissues.

**Table 2:** Heavy metals concentrations in roots and shoots of *P. crassipes* (mg/kg) at different sites of Lake Manzala.

**Heavy metals**		**Site A**	**Site B**	**Site C**	**F- value**
Cu	Root	45.3±2.62^b^	40.3±2.33^ab^	34.6±1.99^a^	5.29***
	Leaf	26.2±1.51^b^	22.8±1.32^a^	27.7±1.59^b^	2.88**
Fe	Root	92.4±5.34^b^	83.6±4.83^b^	60.4±3.49^a^	12.83***
	Leaf	57.6±3.33^a^	53.4±3.08^a^	53.2±3.07^a^	0.62^ns^
Cd	Root	7.8±0.45^b^	7.9±0.46^b^	5.9±0.34^a^	7.23***
	Leaf	5.2±0.30^ab^	5.4±0.31^b^	4.3±0.25^a^	4.14***
Zn	Root	72.3±4.17^b^	79.9±4.61^b^	39.8±2.29^a^	30.94***
	Leaf	61.5±3.55^b^	56.3±3.25^b^	24.8±1.43^a^	46.91***
Pb	Root	27.5±1.59^c^	18.4±1.06^b^	12.5±0.72^a^	41.08***
	Leaf	18.3±1.06^b^	11.9±0.69^a^	9.7±0.56^a^	31.48***
Ni	Root	19.7±1.14^c^	12.9±0.75^b^	9.3±0.54^a^	39.16***
	Leaf	14.2±0.82^b^	8.3±0.48^a^	7.1±0.41^a^	40.50***

Values represent mean ± SE (n = 10). Different superscript letters within the same row indicate significant differences among sites according to Tukey’s test (P < 0.05). ns = not significant; **P < 0.01; ***P < 0.001.

The greatest levels of heavy metals in the below ground roots of *P. crassipes* were Cu, Fe, Pb, and Ni (45.3**±**2.62, 92.4**±**5.34, 27.5**±**0.74, and 19.7**±**1.14 mg/kg, respectively) at site A, Cd and Zn (7.9**±**0.46 and 79.9**±**4.61 mg/kg, respectively) at site B. Conversely, the lowest concentrations of Cu (34.6**±**1.99 mg/kg), Fe (60.4**±**3.49 mg/kg), Cd (5.9**±**0.34 mg/kg), Zn (39.8**±**2.29 mg/kg), Pb (12.5**±**0.72 mg/kg), and Ni (9.3**±**0.54 mg/kg) were detected at site C (Table 2). Furthermore, the greatest levels of Fe, Zn, Pb and Ni were recorded in the aboveground leaves of *P. crassipes* (57.6**±**3.33, 61.5**±**3.55, 18.3**±**1.06, and 14.2**±**0.82 mg/kg, respectively) at the site A, Cu (27.7**±**1.59 mg/ kg) at the site C, Cd (5.4**±**0.31 mg/kg) at the site B. The lowest concentrations of Fe, Cd, Zn, Pb, and Ni (53.2**±**3.07 mg/kg, 4.3**±**0.25 mg/kg, 24.8**±**1.43 mg/kg, 9.7**±**0.56 mg/kg, and 7.1**±**0.41 mg/kg) in leaves of *P. crassipes* were observed at site C. Meanwhile, the lowest levels of Cu (22.8**±**1.32 mg/kg) at site B.

### Relationships between heavy metals

**Fig. 4:**
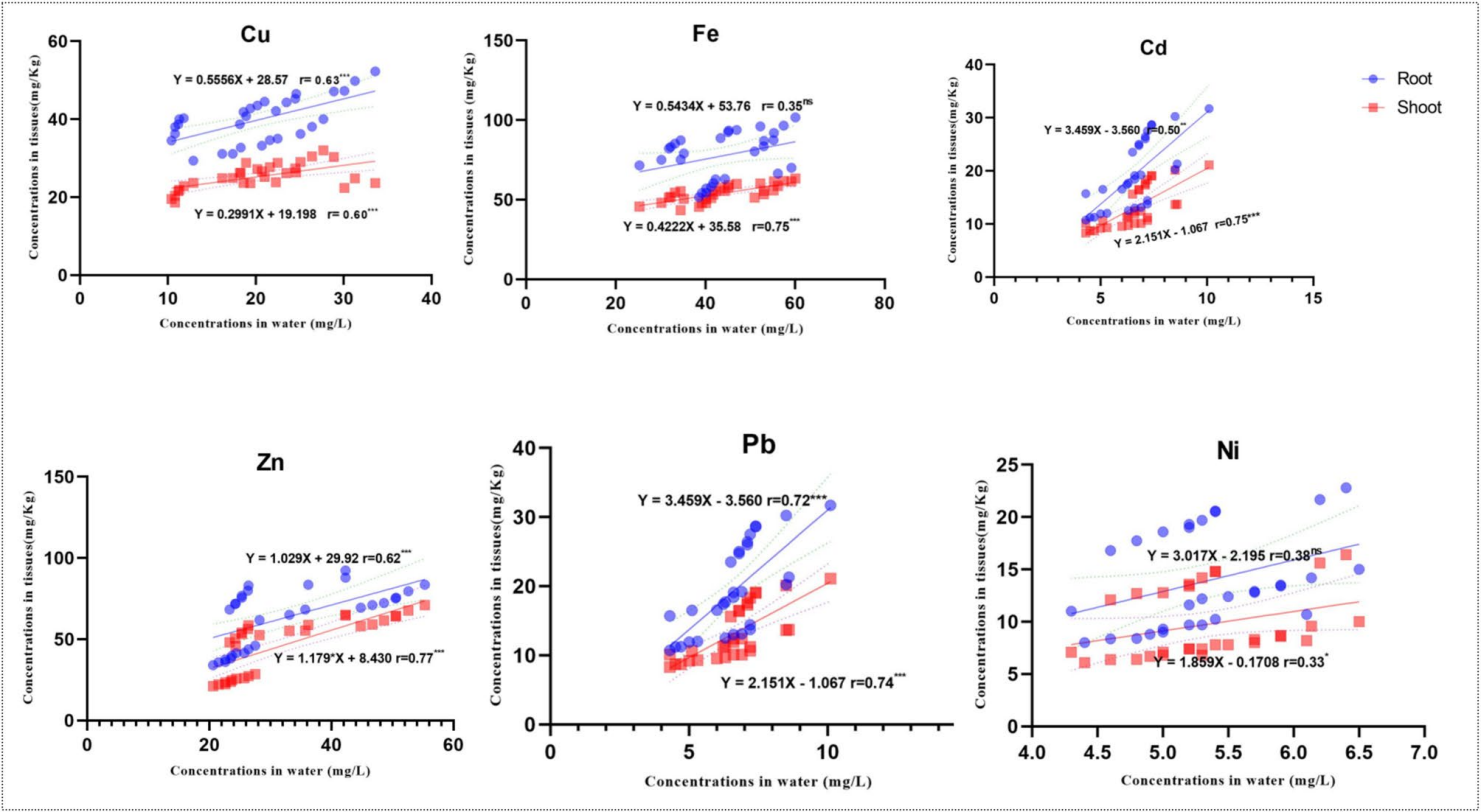
Diagrams of linear regression analysis between heavy metals’ concentrations in water and *P*. *crassipes* roots and leaves.

Regression analysis of heavy metals in the leaves and roots of *P*. *crassipe*s and in water is presented in Fig. 4. Significant positive associations (P < 0.05) were observed between heavy metal concentrations in water and those in the leaves and roots of *P*. *crassipes*. In general, the relationships between metal concentrations in water and those in the leaves were stronger than those observed for the roots, with the exception of Cu and Ni.

Copper concentrations in water showed significant positive correlations with Cu in roots (r = 0.63, P < 0.001) and leaves (r = 0.60, P < 0.001). Iron in water was strongly and positively associated with Fe in the leaves of *P*. *crassipes* (r = 0.75, P < 0.001), whereas its correlation with Fe in roots was positive but not significant (r = 0.35, P > 0.05). Cadmium in water also exhibited significant positive correlations with Cd in leaves (r = 0.75, P < 0.001) and roots (r = 0.50, P < 0.01) (Fig. 4).

Similarly, increasing Zn concentrations in water were associated with increased Zn concentrations in both leaves and roots, with a stronger relationship observed in leaves (r = 0.77) than in roots (r = 0.62). Lead concentrations in water were strongly and positively correlated with Pb levels in both leaves and roots (r = 0.74 and r = 0.72, respectively). In contrast, Ni in water showed a non-significant positive correlation with Ni in roots (r = 0.38) and a weak but statistically significant positive correlation with Ni in leaves (r = 0.33, P < 0.05).

### Bioconcentration factor (BF), biological accumulation coefficient (BAC), translocation factor (TF)

**Fig. 5:**
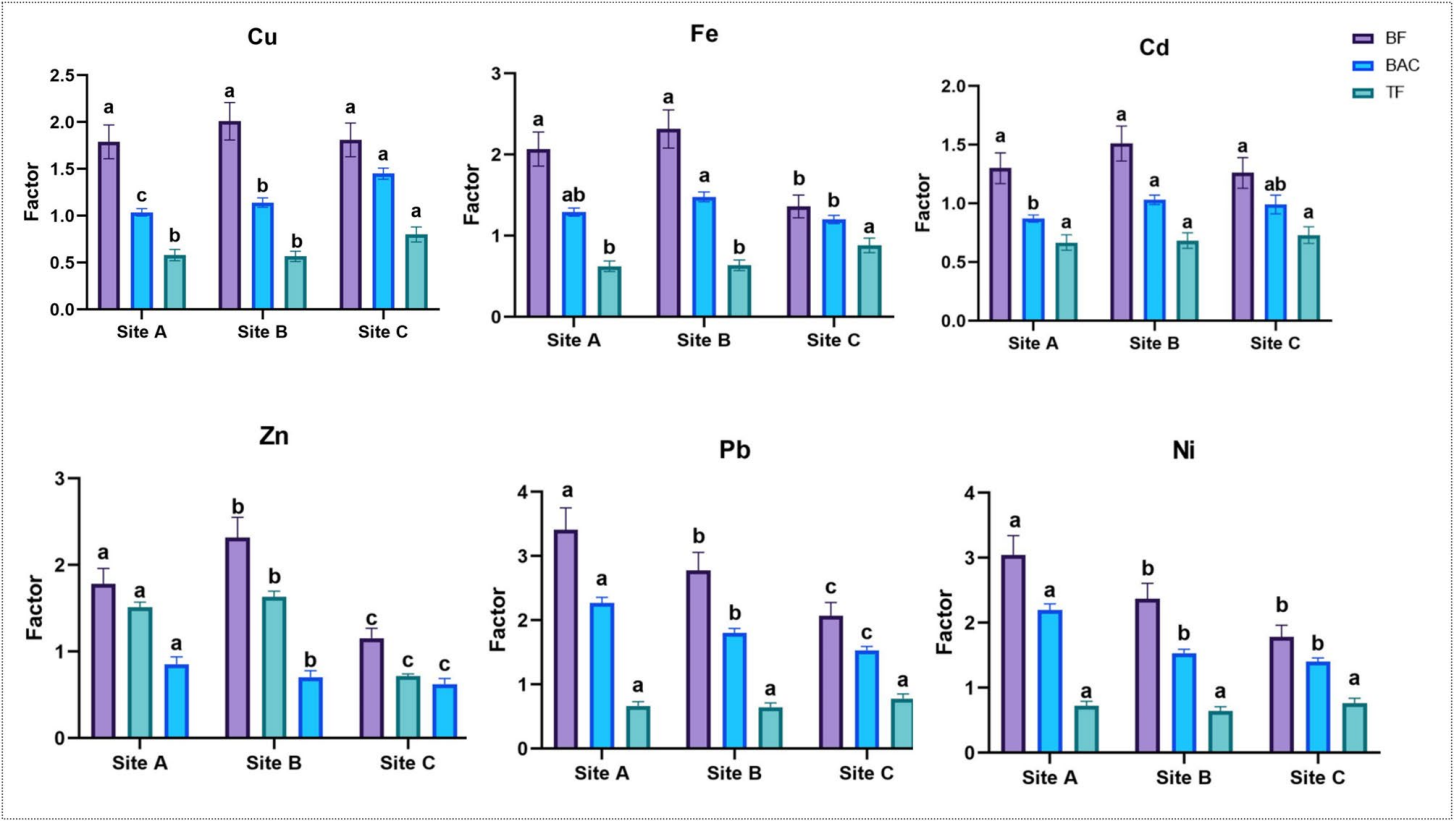
Biological factors (BF), Biological accumulation coefficient (BAC), and Translocation factor (TF) of heavy metals in *P*. *crassipes* grown in Lake Manzala. Different superscript letters indicate significant differences among sites according to one-way ANOVA followed by Tukey’s post hoc test (P < 0.05).

The bioaccumulation potential of *P*. *crassipes* indicated a great variation at studied locations (Fig. 5). The BFs of all heavy metals were more than one at all sites. BF was primarily higher for Pb, followed by Ni, Fe, Cu, Zn and Cd. The highest BF values of Cu, Fe, Cd and Zn were recorded at site B, while BF of Pb and Ni were maximum at site B. However, site C exhibited the least BF values for all metals.

The findings revealed that the BACs of all heavy elements were greater than unity, excluding Cd at site A (BAC =0.87) and Zn at site C (BAC =0.72).

The TF varied between different sites. In general, the TFs for Cu, Fe, Cd, Zn, Pb, and Ni were less than one. The highest TF values (0.80, 0.88, 0.73, 0.78 and 0.76) of Cu, Fe, Cd, Pb, and Ni were recorded at site C, while the highest TF values (0.82) for Zn were recorded at site A. The least TF values of Cu (0.57), Pb (0.65) and Ni (0.64) were at site B. Meanwhile, the lowest TF of Fe (0.62), Cd (0.67) were at site C. TF of Zn (0.62) were the lowest value at site C.

## Discussion

### Spatial variation of heavy metals in water

The present study demonstrated significant spatial variation in heavy metal concentrations across Lake Manzala, with the highest levels consistently recorded at the northeastern site (Site A). The observed order of metal concentrations (Fe > Zn > Cu > Pb > Ni > Cd) reflects both natural geochemical abundance and strong anthropogenic influence. Elevated concentrations at Site A are likely attributed to the continuous inflow of untreated agricultural drainage, industrial effluents, and domestic wastewater through major drainage canals entering the lake.

The modest depth of Lake Manzala and the considerable wind activity on its surface hinder the formation of thermal stratification inside the water body. The highest water temperature was documented at Site A, averaging 31.60°C. The variation in average water temperature readings remained very consistent across several measurement stations^7^. Transparency serves as a sign of water quality, reflecting the extent of light penetration through the water body. The elevated transparency at Sites A and C may be attributed to the greater water depths (193 cm and 217 cm, respectively) at these locations compared to Site B^47^.

Water sample pH varied from 7.84 to 8.76, remaining within the established limits^6,48^. The pH levels were predominantly elevated in northern locations affected by the influx of seawater from inlets. Numerous factors affect total dissolved salts in water, including sewage discharge, seawater intrusion, precipitation, and evaporation^47^. The northeastern region had the greatest total dissolved salts (TDS) levels because of its proximity to the Mediterranean Sea, whereas the middle region displayed the lowest TDS values. Abdo^49^ noted that rises in measured conductivity were associated with similar elevations in total dissolved salts and water temperature.

Dissolved oxygen (DO) is a crucial determinant for sustaining aquatic flora and fauna. Low DO levels were observed near the northern sector of the lake because of the high volume of wastewater inflow and the associated elevated biological oxygen demand^39^. The strong correlation between EC and TDS implied that the dissolved salts were mainly ionic. Significant correlations were found between pH and DO, as well as PO₄^3^⁻, reflecting a state of eutrophication in the lake^39^. The quantities of ammonia were notably elevated at Site A as a result of agricultural waste. Van Loon and Duffy^50^ pointed out that ammonia-containing fertilizers, such as ammonium sulfate, ammonium nitrate, and urea, are major sources of ammonium ions in water. The higher concentrations of phosphate are mostly due to drainage water loaded with phosphorus compounds from agricultural discharges rich in fertilizers^51^.

Silicate is a vital component required for the development of diatoms in coastal waters^52,53^ and significantly influences phytoplankton composition^54,55^. The surplus riverine dissolved inorganic nitrogen (DIN) and dissolved inorganic phosphorus (DIP) from agricultural, industrial, and local wastewater inputs have altered the Si:N:P ratio in recent years^56^. Elevated silicate concentrations were observed in the northern regions of the lake (Sites A and B), and this may be attributed to industrial discharges.

Metals typically enter aquatic environments through geological erosion or anthropogenic activities, including industrial effluents, domestic sewage, and mining waste. Site A showed the highest levels of heavy metals in water, likely due to the polluted sewage, industrial, and agricultural water it receives^26,39,57–59^, whereas Site B had relatively high values within the industrial zone of the Damietta region, and the lowest values were observed in the central area of the lake (Site C).

Similar spatial patterns have been reported in previous studies on Lake Manzala and other Egyptian coastal lakes, where areas receiving drainage inputs exhibit significantly higher metal loads^26,39,60^. Iron and zinc, although essential micronutrients, were found at elevated concentrations that may pose ecological risks when exceeding threshold levels^61^. Cadmium, despite being the least abundant metal, is of particular concern due to its high toxicity and non-essential nature. Its elevated levels in water may result from agricultural fertilizers and industrial discharges, as well as increased mobility under changing physicochemical conditions^62,63^,. These findings confirm that anthropogenic activities are the dominant drivers of heavy metal contamination in Lake Manzala, highlighting the urgent need for improved wastewater management and pollution control strategies.

Copper, iron, and zinc are recognized as essential micronutrients for plants; however, elevated amounts can be highly toxic. They are introduced into the environment through mining and agricultural practices^64^. Cadmium is a toxic, non-essential element and an important ecological contaminant associated with industrial activities, fertilizers, and pesticides^61^. The results of this investigation indicate that water cadmium concentration exceeded the allowable limit established by Kabata-Pendias^65^. This may result from the enhanced mobility of Cd from sediment to water^63^ and its tendency to adsorb onto suspended particulates^62^. Nickel is a necessary trace element for aquatic life but may become hazardous at elevated levels. It enters the environment through both natural weathering of rocks and anthropogenic activities, and more than 90% of nickel in aquatic ecosystems is linked to sedimentary particles^66^. Lead is a non-essential and hazardous heavy metal characterized by high environmental persistence and accumulation potential^67,68^. It is released through extraction and refining processes, as well as industries such as paint, pigments, storage batteries, and gasoline-powered transportation^69^.

### Bioaccumulation behavior of *Pontederia crassipes*

A major finding of this study is the preferential accumulation of heavy metals in the roots of *P*. *crassipes*, where concentrations were significantly higher than in leaves (p < 0.05). This pattern indicates that roots act as the primary site for metal uptake and sequestration. The extensive fibrous root system of *P*. *crassipes* provides a large surface area for adsorption, ion exchange, and complexation processes, facilitating efficient metal accumulation.

This root-dominated accumulation has been widely reported in previous studies on aquatic macrophytes, particularly water hyacinth^70–72^. The high affinity of roots for heavy metals is often attributed to the presence of negatively charged functional groups, such as carboxyl and hydroxyl groups, which bind metal ions^73^. Additionally, intracellular mechanisms such as chelation by phytochelatins and sequestration in vacuoles contribute to metal detoxification and accumulation within root tissues.

The significant positive correlations between metal concentrations in water and plant tissues observed in this study further confirm that *P. crassipes* reflects ambient environmental conditions. This supports its use as a reliable bioindicator of heavy metal pollution, as previously suggested by Bonanno et al.^19^ and Nguyen et al.^20^. Higher accumulation levels at Sites A and B correspond closely with elevated water concentrations, reinforcing the strong dependence of plant uptake on environmental availability.

The concentration of heavy metals in aquatic plants serves as a critical indicator of their bioaccumulation and the associated risks to aquatic organisms and humans^19,20^. The highest concentrations of the analyzed metal ions in *P. crassipes* were observed at Sites A and B, mirroring the distribution of metal content in the water. These findings suggest that the accumulation of heavy metals in plants is strongly dependent on their concentrations in water^60,74^.

Aquatic plants can serve as a natural resource for the remediation of contaminated sites owing to their inherent capacity to absorb heavy metals from water^75–77^. In the present study, water hyacinth not only accumulated various heavy metals but also showed greater accumulation in roots than in leaves. Irrespective of the sampling location, the roots of *P. crassipes* exhibited the highest concentrations of heavy metals, and plants collected from the northeastern region showed greater concentrations than those from other regions.

Heavy metals were predominantly retained in the roots rather than in the leaves, as indicated by translocation factor values below 1^78^. This retention is considered a protective mechanism against metal toxicity, involving both storage of heavy metals in the roots and restriction of their movement to the aerial parts. Sawidis et al.^79^ explained that high metal concentrations in roots may result from adsorption onto the large root surface area and the presence of parenchyma cells. Other studies^71–73,80^ have shown that roots accumulate greater quantities of heavy metals because they are the first plant organs to encounter and absorb metals and because they contain phytochelatins and sulfhydryl groups that support sequestration. Sites A and B, characterized by lower pH values, showed elevated quantities of the examined heavy metals in plant roots. Kashem and Singh^81^ showed that lower pH increases metal availability, which helps explain the higher accumulation of Cd, Cu, Ni, Pb, and Zn under such conditions. The studied species can therefore accumulate high levels of Fe, Cu, Zn, Cd, Ni, and Pb in its belowground parts, making it a potential candidate for remediation of these metals. Previous studies^82–85^, also support our findings that concentrations of Fe, Cu, Cd, Zn, Ni, and Pb in water can serve as explanatory variables for predicting their concentrations in the leaves and roots of *P. crassipes*.

### Phytoremediation potential: BAC, BF, and TF

The phytoremediation potential of *P. crassipes* was evaluated using bioconcentration factor (BF), biological accumulation coefficient (BAC), and translocation factor (TF). BF and BAC values exceeding 1 for most metals indicate a strong capacity of the plant to accumulate heavy metals from water, confirming its efficiency as a bioaccumulator. These findings are consistent with previous studies demonstrating high accumulation efficiency of *P. crassipes* for metals such as Cu, Pb, Zn, and Cd^15–17^. High BF values suggest that the plant can effectively remove metals from contaminated water, making it suitable for phytoremediation applications. In contrast, TF values were consistently below 1, indicating limited movement of metals from roots to aerial parts. This suggests that *P. crassipes* primarily functions as a phytostabilizer rather than a phytoextractor. According to established criteria, plants with BF > 1 and TF < 1 are considered effective for phytostabilization because they immobilize contaminants within root tissues and reduce their mobility in the environment^86–88^.

The restricted translocation observed in this study may represent an adaptive mechanism to minimize metal toxicity by preventing excessive accumulation in metabolically active aerial tissues. Similar findings have been reported by Sharma et al.^78^, who noted that aquatic plants often retain heavy metals in roots as a protective strategy. According to our data, the majority of metals exhibited BF and BAC values greater than 1, whereas TF values were below 1 because many metals were concentrated in the roots. These TF values indicate a reduced rate of metal transfer from belowground tissues to aboveground tissues.

### Environmental implications and comparison with previous studies

The results of this study agree with previous research indicating that *P. crassipes* is highly effective in accumulating heavy metals and can serve both as a bioindicator and a phytoremediator in polluted aquatic systems^70,84,85^. However, unlike many earlier studies conducted under controlled conditions or limited spatial scales, this study provides a field-based, multi-site assessment within a large and heterogeneous lake system. The integration of BAC, BF, and TF in a spatial framework represents an important advancement in evaluating phytoremediation efficiency under natural environmental conditions. This approach allows a more comprehensive understanding of metal uptake dynamics and the ecological function of aquatic macrophytes. From an environmental management perspective, the high accumulation capacity and widespread distribution of *P. crassipes* make it a practical candidate for large-scale phytoremediation programs. However, its invasive nature should also be considered, and proper management strategies are required to balance its ecological benefits with potential risks.

## Conclusion

This study demonstrated significant spatial variation in heavy metal contamination across Lake Manzala, with the highest concentrations recorded at sites influenced by agricultural, industrial, and domestic discharges. The overall metal concentration pattern in water followed the order: Fe > Zn > Cu > Pb > Ni > Cd, indicating substantial anthropogenic impact on the lake ecosystem. *Pontederia crassipes* exhibited a strong capacity for heavy metal accumulation, with significantly higher concentrations detected in roots compared to leaves. The calculated bioconcentration factor (BF) and biological accumulation coefficient (BAC) values greater than 1 confirm its high accumulation efficiency, while translocation factor (TF) values below 1 indicate limited transfer of metals to aerial parts. These results suggest that the species primarily functions as a phytostabilizer by immobilizing heavy metals within root tissues. The significant relationships between metal concentrations in water and plant tissues further support the suitability of *P. crassipes* as a reliable bioindicator of heavy metal pollution. Overall, the findings highlight the dual role of *P. crassipes* in environmental monitoring and phytoremediation. Its application can contribute to sustainable management strategies aimed at reducing heavy metal contamination and improving water quality in Lake Manzala and similar freshwater ecosystems.

The above results verified that the lake is confronting a significant risk of metal contamination. The primary source of metal contamination in wastewater effluents arises from industrial, household, and agricultural pollutants, particularly in the northeastern region of the Lake, which can deteriorate water quality, induce eutrophication, and modify phytoplankton communities. *P*. *crassipes* (water hyacinth) is a prevalent aquatic plant in numerous tropical nations. Its capacity to remove heavy metals from water has enabled its usage for purifying reasons. The absorption of heavy metals in this plant is more pronounced in the roots than in the floating leaves. Its capacity to absorb elevated concentrations of metals renders it an effective biomonitor for heavy metal pollution in Lake Manzala.

## Data Availability

All recorded, measured, and analyzed datasets (e.g., independent measurements of the five biological and technical replicates) were completely incorporated within the manuscript’s main text.
